# Adjuvant treatment combining cellular immunotherapy with chemotherapy improves the clinical outcome of patients with stage II/III gastric cancer

**DOI:** 10.1002/cam4.942

**Published:** 2016-10-27

**Authors:** Yizhuo Wang, Chang Wang, Huijie Xiao, Chao Niu, Haitao Wu, Haofan Jin, Cheng Yao, Hua He, Huimin Tian, Fujun Han, Dan Li, Wei Han, Jianting Xu, Jingtao Chen, Jiuwei Cui, Wei Li

**Affiliations:** ^1^ Cancer Centerthe First Hospital of Jilin UniversityChangchunChina; ^2^ Department of Gastrointestinal Colorectal and Anal SurgeryChina–Japan Union Hospital of Jilin UniversityChangchunChina; ^3^ Institute of Translational Medicinethe First Hospital of Jilin UniversityChangchun130031China

**Keywords:** Cellular immunotherapy, chemotherapy, disease‐free survival, gastric cancer, overall survival

## Abstract

Postsurgical relapse remains a common issue for resectable gastric cancer (GC). Here, we investigated the efficacy and safety of an adjuvant treatment combining chemotherapy with cellular immunotherapy (CIT) using autologous natural killer cells, *γ*δT cells, and cytokine‐induced killer cells in the treatment of stage II/III GC. A pilot prospective cohort study was conducted in 169 patients with stage II/III GC who had undergone gastrectomy with D2 lymph node dissection. Patients were assigned into two groups according to the patient choice of treatment, including chemotherapy alone (chemo) or chemotherapy combined with CIT (chemo/CIT). Disease‐free survival (DFS), overall survival (OS), and adverse events were evaluated. Univariate and multivariate Cox models were used to analyze the impact of chemo/CIT on DFS and OS. Kaplan–Meier analysis with the log‐rank test was used to compare the clinical outcome between two groups. Three‐year DFS rate was 60.6% and 74.7% (*P *=* *0.036) and 3‐year OS rate was 64.9% and 83% (*P *=* *0.051) for the chemo and chemo/CIT group, respectively. TNM stage and chemo/CIT were independent prognostic factors for both DFS (for TNM stage, *P *<* *0.001, hazard ratio [HR]: 5.599, 95% confidence interval [CI]: 2.791–11.232; for chemo/CIT, *P *=* *0.013, HR: 0.478, 95% CI: 0.266–0.858) and OS (for TNM stage, *P *<* *0.001, HR: 6.559, 95% CI: 2.903–14.817; for chemo/CIT, *P *=* *0.04, HR: 0.506, 95% CI: 0.264–0.970). In subgroup analysis, 3‐year DFS and OS rates of patients with stage III GC in the chemo/CIT group were significantly higher than those in the chemo group (38.4% vs. 57.1%, *P *=* *0.038; and 45.9% vs. 76%, *P *=* *0.06, respectively), while there was no significant difference between the two groups in patients with stage II GC. Only 15.9% of patients (10/63) in the chemo/CIT group had mild and manageable fever (grades 1 and 2), while no other side effects were observed. The adjuvant treatment combining chemotherapy with cellular immunotherapy is well tolerated and significantly improves the clinical outcome of patients with stage II/III GC, when compared with chemotherapy alone, therefore warrants further attention in treatment for relapsed GC after tumor resection.

## Introduction

Gastric cancer (GC) is the fifth most common malignancy and the fourth leading cause of cancer‐related death worldwide. According to GLOBOCAN, almost one million new cases and over 720,000 deaths were estimated in 2012 1. Almost two thirds of these cases occur in developing countries, while 42% in China 1. Surgical resection is the cornerstone for the treatment of patients with localized gastric cancer. However, postsurgical relapse occurs in approximately 40% of patients within 2 years after surgery 2. Therefore, there has been much interest in using adjuvant therapies to improve the outcomes of GC patients after surgical resection of the primary tumor. Unfortunately, although various strategies have been attempted, few have shown to be clinically beneficial 3, 4, 5, 6, 7, 8. So far, there are only two large randomized phase III studies (i.e., the ACTS GC trial 9 and the CLASSIC trial 10) in Asia that have demonstrated some survival benefit of postoperative chemotherapy following curative D2 lymph node dissection in patients with resectable GC, which therefore support the use of chemotherapy as the standard care for the treatment of these patients after surgery. However, due to high recurrence of tumor, novel therapeutic regimens are urgently needed to improve the efficacy of postoperative adjuvant chemotherapy and thereby clinical outcomes of patients with GC after surgical resection of primary tumor.

Bulk evidence obtained from *in vitro* and *in vivo* studies has revealed that surgery as well as GC itself could impair the function of immune system. For example, postoperative changes in the systemic immune response are associated with the degree of surgical trauma, which might contribute to the development of septic complications and tumor metastasis 11. On the other hand, activity of immunocytes is often compromised in GC patients 12. Furthermore, tumor cells can also downregulate major histocompatibility complex (MHC) molecules 13, and thereby protect themselves from recognition and clearance via the immune system (e.g., by cytotoxic T lymphocytes, CTLs) 14. To this end, adoptive cellular immunotherapy (CIT) using non‐MHC‐restricted immune cells (e.g., NK [natural killer], *γ*δT, and CIK [cytokine‐induced killer] cells) has been introduced as an approach to enhance the function of immune system in patients with cancer. Of note, while NK, *γ*δT, and CIK cells function in a similar manner against tumor cells, they exert synergistic effects in killing tumor cells when used in combination 15, suggesting that the combined use of NK, *γ*δT, and CIK cells might improve therapeutic activity of cellular immunotherapy. Interestingly, current adjuvant chemotherapy for treatment of GC patients is based on 5‐fluorouracil in combination with oxaliplatin, which is known to upregulate the expression of natural killer group 2, member D (NKG2D) in tumor cells, and thereby increase their susceptibility to NK, *γ*δT, or CIK cell‐mediated killing 16. Moreover, CIT cells have been shown to improve the efficacy of chemotherapy 17.

It remains to be defined whether CIT would be effective in the adjuvant treatment of patients with local advanced GC. Therefore, this prospective cohort study was designed and conducted to investigate the efficacy and safety of an adjuvant regimen combining chemotherapy with CIT as a novel therapeutic strategy to improve the clinical outcome of patients with stage II/III GC. Here, we report that adjuvant chemotherapy combined with CIT is well tolerable and significantly improves the clinical outcome of patients with stage II/III GC after radical surgery.

## Methods

### Patients and study design

This is a prospective and observational cohort study approved by the institutional review board of the First Hospital of Jilin University. Patients diagnosed with stage II/III gastric cancer after undergoing a gastrectomy with D2 lymph node dissection were enrolled between 2010 and 2012 at the First Hospital of Jilin University. All patients provided informed consent to participate in this study. Eligible patients met the following criteria: (1) ≥18 years old, (2) diagnosis as stage II/III gastric cancer, with a primary gastric tumor pathologically diagnosed as adenocarcinoma, (3) no more than 4 weeks after a gastrectomy with D2 lymph node dissection, (4) Eastern Cooperative Oncology Group (ECOG) performance status ≤2, and (5) normal liver, renal, and hematologic functions. The World Health Organization Classification of Tumors was used for histological grading and the TNM classification of the American Joint Committee on Cancer (AJCC) was used for tumor staging 18. Exclusion criteria included: (1) other immunotherapies, (2) severe infections, (3) a history of organ transplantation, and (4) pregnancy or breastfeeding.

All the enrolled patients were advised to receive standard 5‐fluorouracil‐ and platinum‐based adjuvant chemotherapy to reduce the risk of recurrence according to the National Comprehensive Cancer Network (NCCN) Guidelines 19. According to the patient choice of treatment, patients were assigned into two groups: chemotherapy alone (chemo) or combined chemotherapy and CIT (chemo/CIT). Patients were followed up every 3 months postoperatively, including complete physical examination, analysis of basic serum chemistry, and contrast‐enhanced computed tomography (CT) of the chest and abdomen.

The primary outcome of this study is disease‐free survival (DFS). The secondary outcomes include overall survival (OS) and systemic side effects of CIT. DFS was defined as the length of time from the date of surgery to the date of progression or to the date of the most recent follow‐up. OS was defined as the length of time from the date of surgery to the date of death or to the date of the most recent follow‐up. Survival status was last updated in August 2015.

### Preparation of immunocytes

Peripheral blood mononuclear cells (PBMCs, 1–1.5 × 10^9^ cells) were collected from each patient 1–2 days before adjuvant chemotherapy using Spectra Optia^®^ Apheresis System (Gambro BCT, Inc, Lakewood, CO) as per manufacturer's instructions. After collection, PBMCs was split into two 50‐mL centrifuge tubes that were spun for 10 min at 1734g. The supernatant was discarded and the cell pellets were resuspended in 30 mL of phosphate‐buffered saline (PBS) and placed on top of a 15‐mL Hypaque (Amersham Biosciences, Uppsala, Sweden) in a 50‐mL sterile tube. Lymphocytes were isolated from PBMCs by means of Ficoll‐Hypaque density centrifugation (Ficoll separation) to yield ~1.3 × 10^9^ (1.0–1.8 × 10^9^) PBMCs. Cells were then separated into three pools to induce NK, *γ*δT, and CIK cells through the use of different cytokines as described previously 20. All procedures for preparing the autologous immune cells were carried out under good manufacturing practice conditions approved by the Jilin Provincial Center for Sanitation Inspection and Test (China, certificate ID A20090047).

### Characterization of immunocytes

Immunocytes for infusion were characterized using fluorescence‐conjugated primary monoclonal antibodies against specific cell surface markers (NK, CD3^−^/CD56^+^; *γ*δT, V*γ*9^+^; CIK, CD3^+^/CD56^+^) by four‐color FACSCalibur flow cytometry (BD Biosciences, San Diego, CA) as described previously 20, 21.

### Infusion of immunocytes

**Figure 1:**
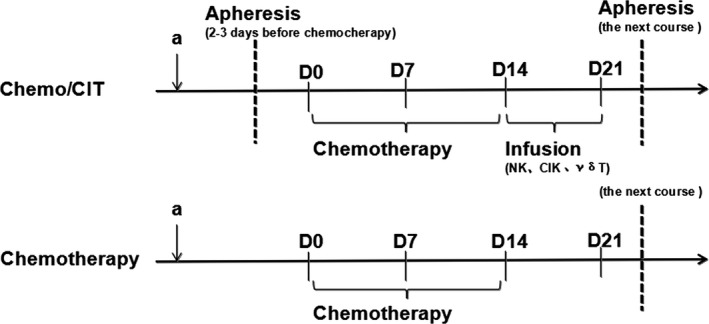
Treatment schedule of chemotherapy and chemoimmunotherapy.

NK, *γ*δT, and CIK cells were washed three times with normal saline and individually resuspended in 50 mL normal saline. Within half an hour, cells were then administered via an intravenous drip. The number of cells for each infusion ranged from 1.2 to 2.0 × 10^9^ cells. A course of CIT was completed within 3 weeks, with an infusion focus within 14–21 days, after apheresis. For each course, eight sessions of infusion were performed. Collection of PBMCs for the next course started 1–2 days before the last infusion of the previous course. The patients in the chemo/CIT group received continuous treatment with six courses of CIT. The schedule for treatment is shown in Figure 1.

### Assessment of treatment‐related side effects

All adverse events experienced by the patients were recorded and graded according to the Common Terminology Criteria for Adverse Events (CTCAE) Version 4.0 22.

### Statistical analysis

The clinical characteristics were compared between the chemo and the chemo/CIT groups using the chi‐square test. DFS and OS curves were estimated by the Kaplan–Meier analysis, and compared between two groups using the Cox's model for hazard ratio (HR) and 95% confidence interval (CI). Data of immunocyte phenotypes were recorded as the mean ± standard deviation (SD). Percentages of lymphocytes were compared between before and after CIT using the paired sample *t*‐test. The degree of myelosuppression was compared between two groups using the rank‐sum test. An SPSS software (Version 17.0; SPSS, Inc., Chicago, IL) was used for all statistical analyses. *P *<* *0.05 was considered statistically significant.

## Results

### Patient characteristics

A total of 169 patients were enrolled in this prospective cohort study. Six patients in the chemo group and four patients in the chemo/CIT group were excluded due to loss to follow‐up. As consequence, a total of 159 patients, including 96 patients in the chemo group and 63 in the chemo/CIT group, were subjected to analysis. Patients in these two groups had similar demographics (Table 1). There was no significant difference in sex, age, as well as tumor location, differentiation, and stage between two groups (*P *>* *0.05 for each case). Median follow‐up time was 48.6 months (range: 16.3–69.6 months).

**Table 1:** Patient characteristics

	No. of patients	Adjuvant therapy, *n* (%)		*P* value
		Chemo (%)	Chemo/CIT (%)	
Sex				
Male	123	73 (59.3)	38 (40.7)	0.624
Female	36	23 (63.9)	13 (36.1)	
Age (years)				
<60	97	61 (62.9)	36 (37.1)	0.418
≥60	62	35 (56.5)	27 (43.5)	
Stage (AJCC)				
II	69	41 (59.4)	28 (40.6)	0.892
III	90	55 (61.1)	35 (39.6)	
Location				
GEJ	30	14 (46.7)	16 (53.3)	0.088
NGEJ	129	82 (63.6)	47 (36.4)	
Differentiation				
Intermediate	59	33 (55.9)	26 (44.1)	0.379
Poor	100	63 (63.0)	37 (37.0)	

AJCC, American Joint Committee on Cancer.

### Quality of immunocytes

**Figure 2:**
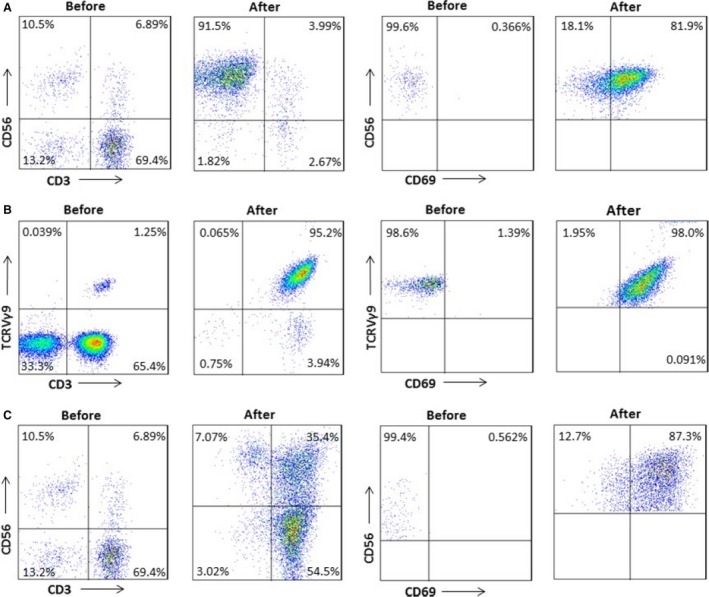
Characterization of NK, *γ*δT, and CIK cells before and after induction. Results from one representative patient are shown. Percentage of activated NK cells (A), *γ*δT cells (B), and CIK cells (C) before and after induction was 0.366% versus 81.9%, 1.39% versus 98%, and 0.562% versus 87.3%, respectively.

The percentages of NK (CD3^−^/CD56^+^), CIK (CD3^+^/CD56^+^), and *γ*δT (V*γ*9^+^) cells before and after induction were 11.94% (8.06–15.56%) versus 80.17% (63.2–93.5%), 9.38% (6.39–13.04%) versus 34.4% (27.83–45.71%), and 2.886% (1.07–4.01%) versus 69.66% (50.23–84.01%), respectively (Table 2). Results from a representative patient are shown in Figure 2. Viability of immunocytes exceeded 95%.

**Table 2:** Percentage of NK, *γ*δT, and CIK cells before and after induction

	Before (median, range) (%)	After (median, range) (%)
NK cells	11.9 (8.1–15.6)	80.2 (63.2–93.5)
*γ*δT cells	2.9 (1.1–4.0)	69.7 (50.2–84.0)
CIK cells	9.4 (6.4–13.0)	34.4 (27.8–45.7)

CIK, cytokine‐induced killer.

### Clinical outcomes

**Figure 3:**
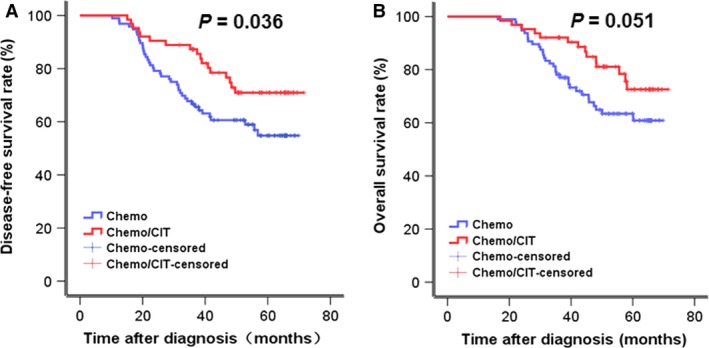
Clinical outcomes of patients who received chemotherapy or chemotherapy plus CIT. Kaplan‐Meier survival analysis was used to compare three‐year DFS and OS rate between the chemo and the chemo/CIT group (DFS, 60.6% vs 74.7%, *P* = 0.036; OS, 64.9% vs 83%, *P* = 0.051).

To examine efficacy of adjuvant chemotherapy combined with CIT in patients with GC, the Kaplan–Meier analysis was performed to compare DFS and OS rates between the chemo and the chemo/CIT group in a total of 159 assessable patients. As shown in Figure 3, adjuvant treatment combining chemotherapy and CIT significantly increased 3‐year DFS rate (74.7% vs. 60.6%, *P *=* *0.036) and marginally significantly 3‐year OS rate (83% vs. 64.9%, *P *=* *0.051), compared to chemotherapy alone.

**Figure 4:**
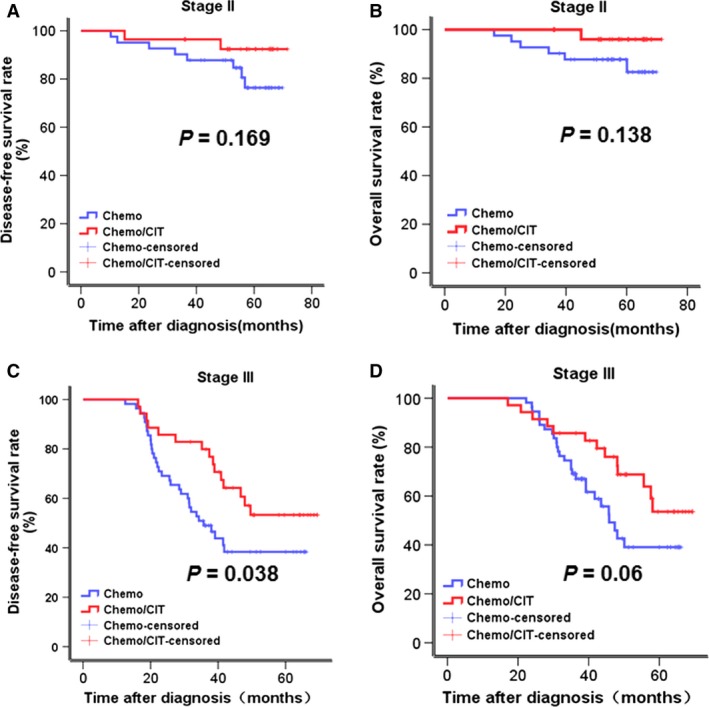
Kaplan–Meier survival analysis was used to compare 3‐year DFS and OS rate between the chemo and the chemo/CIT groups (DFS, 60.6% vs. 74.7%, *P* = 0.036; OS, 64.9% vs. 83%, *P* = 0.051). Subgroup analysis of clinical outcomes of patients who received chemotherapy or chemotherapy plus CIT. Three‐year DFS and OS rates were analyzed in patients with stage II (chemo vs. chemo/CIT: DFS, 87.7% vs. 92.4%, *P* = 0.169; OS, 87.7% vs. 96%, *P* = 0.138) and III (chemo vs. chemo/CIT: DFS, 38.4% vs. 57.1%, *P* = 0.038; OS, 45.9% vs. 76%, *P* = 0.06), respectively.

In subgroup analysis (Fig. 4), 3‐year DFS and OS rates of patients with stage II GC in the chemo group were moderately but not significantly lower than those in the chemo/CIT group (DFS, 87.7% vs. 92.4%, *P *=* *0.169; OS, 87.7% vs. 96%, *P *=* *0.138). However, in patients with stage III GC, 3‐year DFS rate for the chemo/CIT group were significantly higher than those in the chemo group (57.1% vs. 38.4%, *P *=* *0.038), while the difference for OS rate was marginally significant (76% vs. 45.9%, *P *=* *0.06).

### Analysis of prognostic factors

To estimate the effects of adjuvant chemotherapy combined with CIT on postoperative prognosis of GC patients, univariate Cox regression analyses on DFS (Table 3) and OS (Table 4) were performed in this study cohort. Of note, the results revealed that treatment with combination of chemotherapy and CIT was a significant factor on DFS and OS (HR: 0.549, 95% CI: 0.311–0.970; HR: 0.541, 95% CI: 0.289–1.013, respectively). In addition, TNM stage represented another significant factor (DFS, HR: 5.282, 95% CI: 2.653–10.516; OS, HR: 6.267, 95% CI: 2.794–14.057).

**Table 3:** Univariate and multivariate Cox regression analyses of disease‐free survival

	Univariate Cox regression		Multivariate Cox regression	
	HR	95% CI	HR	95% CI
Sex				
Female	1.00	–	1.00	–
Male	1.988	0.942–4.199	2.417	1.132–5.161
Age (years)				
<60	1.00	–	1.00	–
≥60	0.848	0.495–1.453	0.867	0.502–1.497
Stage (AJCC)				
II	1.00	–	1.00	–
III	5.282	2.653–10.516	5.599	2.791–11.232
Location				
NGEJ	1.00	–	1.00	–
GEJ	1.309	0.705–2.430	1.095	0.575–2.084
Differentiation				
Intermediate	1.00	‐	1.00	–
Poor	1.586	0.899–2.799	1.486	0.832–2.655
Adjuvant therapy				
Chemo	1.00	‐	1.00	–
Chemo/CIT	0.549	0.311–0.970	0.478	0.266–0.858

AJCC, American Joint Committee on Cancer; CIT, chemotherapy with cellular immunotherapy.

**Table 4:** Univariate and multivariate Cox regression analyses of overall survival

	Univariate Cox regression		Multivariate Cox regression	
	HR	95% CI	HR	95% CI
Sex				
Female	1.00	–	1.00	–
Male	1.812	0.812–4.045	0.889	0.484–1.634
Age (years)				
<60	1.00	–	1.00	–
≥60	0.842	0.464–1.528	0.889	0.484–1.634
Stage (AJCC)				
II	1.00	–	1.00	–
III	6.267	2.794–14.057	6.559	2.903–14.817
Location				
NGEJ	1.00	–	1.00	–
GEJ	1.206	0.599–2.426	1.008	0.485–2.094
Differentiation				
Intermediate	1.00	–	1.00	–
Poor	1.979	1.027–3.813	1.938	0.990–3.792
Adjuvant therapy				
Chemo	1.00	–	1.00	–
Chemo/CIT	0.541	0.289–1.013	0.506	0.264–0.970

AJCC, American Joint Committee on Cancer; CIT, chemotherapy with cellular immunotherapy.

Furthermore, multivariate Cox regression analysis was performed to evaluate the robustness of the prognostic values of adjuvant chemotherapy combined with CIT or TNM stage. As shown in Tables 3 and 4, treatment with combination of chemotherapy and CIT (DFS, HR : 0.478, 95% CI: 0.266–0.858; OS, HR: 0.506, 95% CI: 0.264–0.970) and TNM stage (DFS, HR: 5.599, 95% CI: 2.791–11.232; OS, HR: 6.559, 95% CI: 2.903–14.817) were independent prognostic factors of DFS and OS, respectively.

### Safety

In the chemo/CIT group, 10 (15.9%) of 63 of patients had grades 1 and 2 fever and shivering after received CIT, which were managed within 2 h by administration of antipyretics. No other CIT‐related adverse effects of were observed. Interestingly, bone marrow suppression occurred modestly less in the chemo/CIT group than the chemo group (52% vs. 59%, *P *=* *0.675, Table 5).

**Table 5:** Bone marrow suppression in the chemo and the chemo/CIT group

Degree	Intervention, *n* (%)		*P* value (for I–IV)
	Chemo	Chemo/CIT	
0	39 (40.6%)	30 (47.6%)	
I+II	39 (40.6%)	22 (34.9%)	0.675
III+IV	18 (18.8%)	11 (17.5%)	

## Discussion

Postoperative chemotherapy could improve clinical outcome of GC patients who have received curative surgery with D2 lymph node dissection, while its efficacy is comprised by high recurrence and metastasis. The latter is closely associated with immunosuppression in patients with GC 23. In this prospective cohort study, it was observed that adjuvant treatment combining chemotherapy and CIT increased significantly DFS rate, as well as marginally significantly OS rate, of patients with stage II/III GC, when compared to chemotherapy alone, suggesting that this approach may represent a promising treatment to further improve clinical outcome of GC patients after primary tumor resection.

Although a recent clinical study has shown that CIT is effective against GC 24, CIT has however not used as a routine treatment for patients with GC, primarily due to limited evidence of efficacy. The benefits of combining chemo‐ and immunotherapy after surgery may stem from restoration of immune function in GC patients often with immunosuppression because of curative resection of primary tumor, or synergistic effects between chemo‐ and immunotherapy, or both. For example, combining chemotherapy with OK‐432, a Streptococcus‐derived immunotherapeutic agent, is more effective than chemotherapy alone against GC 25. Moreover, a phase III clinical trial has revealed that chemoimmunotherapy significantly prolongs recurrence‐free survival and OS rates of patients with resectable GS, compared to chemotherapy alone 26.

The present study reveals that combined chemotherapy with CIT of NK, *γ*δT, and CIK cells significantly reduced the risk of stage II/III gastric cancer recurrence and metastasis compared with chemotherapy alone, manifested by increased DFS rate. Consistently, OS rate for combined treatment showed a similar tendency, although marginally significant, when compared to chemotherapy alone. Moreover, univariate and multivariate regression analyses of DFS and OS further demonstrate that in this cohort of patients with stage II/III GC, combined chemotherapy with CIT was a more beneficial postoperative adjuvant treatment than chemotherapy only.

Subgroup analysis suggests that patients with stage III GC might benefit more from combination therapy of chemotherapy with CIT than those with stage II GC. Similarly, other studies have also shown that a subset of patients with T3/T4a, N2, or stage III preferably benefit from chemoimmunotherapy 26, 27. One possibility is that immunosuppression might be worse in patients with advanced than early‐stage GC, thereby suggesting that the former may be more susceptible to chemoimmunotherapy.

In this study, after patients received six courses of CIT, only ~16% patients experienced mild and manageable fever, while no other CIT‐related adverse event was observed. These findings support a notion that combining chemotherapy and CIT as adjuvant treatment is well tolerated in patients with GC. Of note, myelosuppression occurred less after treated with chemotherapy in combination with CIT than itself alone, suggesting that addition of CIT might provide more benefits (e.g., reducing chemotherapy‐associated myelosuppression) to patients with GC.

However, there are some limitations in the present study. First, while the maximal six courses of CIT were administrated in this pilot study, it remains to be defined whether further increases in course number and timing of CIT would yield better efficacy of CIT in combination with chemotherapy as postoperative treatment of patients with GC. Thus, future clinical trials designed specifically to determine optimal dosing and schedule of CIT are required to address this issue. Second, although this is a perspective study, assignment of patients into either control (chemotherapy alone) or intervention (chemotherapy plus CIT) group was based on the patient choice of treatment, rather random, suggesting a possibility of selection bias. Therefore, although such a study design was due to a consideration from the standpoint of the current clinical practice, future randomized control trials are required to consolidate the findings of this study. Finally, OS was designed as the secondary, rather primary, outcome because of (1) a relatively short period of follow‐up and (2) varied treatments (e.g., chemotherapy, radiotherapy, chemoradiotherapy, or even no treatment) after tumor recurrence, which influence OS of patients.

In summary, the present findings indicate that combining chemotherapy with CIT after radical surgery improves clinical outcome (e.g., DFS and OS) of patients with advanced GC, especially those with stage III disease, compared to chemotherapy alone. This regimen was well tolerated, while might provide potentially more benefits to patients (e.g., reducing occurrence of chemotherapy‐associated myelosuppression). Therefore, this study lays a foundation for combining chemotherapy and CIT as an adjuvant treatment of GC patients after surgery. Consequently, efforts to develop this regimen further in randomized clinical trials of GC are currently underway.

## Conflict of Interest

All authors disclose no commercial, proprietary, or financial interest in the products or companies described in this article.
